# Effects of Temperature and Random Forces in Phase Transformation of Multi-Stable Systems †

**DOI:** 10.3390/e26121109

**Published:** 2024-12-18

**Authors:** Giuseppe Florio, Stefano Giordano, Giuseppe Puglisi

**Affiliations:** 1 Department of Civil, Environmental, Land, Building Engineering and Chemistry (DICATECh), Polytechnic University of Bari, Via Orabona 4, 70125 Bari, Italy; giuseppe.puglisi@poliba.it; 2 INFN, Section of Bari, 70126 Bari, Italy; 3 University of Lille, CNRS, Centrale Lille, Université Polytechnique Hauts-de-France, UMR 8520-IEMN-Institut d’Électronique de Microélectronique et de Nanotechnologie, F-59000 Lille, France; stefano.giordano@univ-lille.fr

**Keywords:** random forces, multi-stable systems, replica trick, Ising model in a random field

## Abstract

Multi-stable behavior at the microscopic length-scale is fundamental for phase transformation phenomena observed in many materials. These phenomena can be driven not only by external mechanical forces but are also crucially influenced by disorder and thermal fluctuations. Disorder, arising from structural defects or fluctuations in external stimuli, disrupts the homogeneity of the material and can significantly alter the system’s response, often leading to the suppression of cooperativity in the phase transition. Temperature can further introduce novel effects, modifying energy barriers and transition rates. The study of the effects of fluctuations requires the use of a framework that naturally incorporates the interaction of the system with the environment, such as Statistical Mechanics to account for the role of temperature. In the case of complex phenomena induced by disorder, advanced methods such as the replica method (to derive analytical formulas) or refined numerical methods based, for instance, on Monte Carlo techniques, may be needed. In particular, employing models that incorporate the main features of the physical system under investigation and allow for analytical results that can be compared with experimental data is of paramount importance for describing many realistic physical phenomena, which are often studied while neglecting the critical effect of randomness or by utilizing numerical techniques. Additionally, it is fundamental to efficiently derive the macroscopic material behavior from microscale properties, rather than relying solely on phenomenological approaches. In this perspective, we focus on a paradigmatic model that includes both nearest-neighbor interactions with multi-stable (elastic) energy terms and linear long-range interactions, capable of ensuring the presence of an ordered phase. Specifically, to study the effect of environmental noise on the control of the system, we include random fluctuation in external forces. We numerically analyze, on a small-size system, how the interplay of temperature and disorder can significantly alter the system’s phase transition behavior. Moreover, by mapping the model onto a modified version of the Random Field Ising Model, we utilize the replica method approach in the thermodynamic limit to justify the numerical results through analytical insights.

## 1. Introduction

The study of the effects of fluctuations and randomness in materials, for instance due to unavoidable interaction of the sample with the environment or because of the presence of defects, is an important area of research in material science both theoretical and technological [1,2,3,4,5,6,7,8,9,10,11,12]. Extensive research activities in this field is driven by the possibility to uncover new phenomena and improve the capability to design innovative materials with improved performance and novel functionalities. In particular, disorder can play a fundamental role in determining physical properties of materials, also influencing the electronic behavior and the response to mechanical action. These effects are particularly pronounced in systems where the assumptions of perfect (crystalline) order is no longer valid, or where external stimuli are subject to random fluctuations, leading to complex behaviors that are challenging to predict. Thus, the comprehension and description of the effects of disorder are of paramount importance to comprehend and control the behavior of a material under various real-world physical conditions or design devices with innovative properties.

Phase transformations, phase transitions, and configurational transformations, such as the passage among different crystal structures, change from a solid to a liquid phase or in folded/unfolded conformational changes in macromolecules, are typically influenced by external actions, including mechanical stress, thermal fluctuations, and internal environmental heterogeneities. As a matter of fact, fluctuations and disorder can lead to deviations from the ideal behavior introducing complexities that are crucial for both theoretical models and practical applications. They induce modifications in materials, also affecting phenomena such as nucleation and growth [13,14]. For instance, in solid-state phase transformations, thermal fluctuations play a fundamental role in nucleation processes where a new phase forms in a small region of the material. The energy barrier for nucleation is typically high, but thermal fluctuations can provide the necessary energy to overcome it [15]. In crystalline materials, disorder can manifest as point defects or dislocations, thus altering the periodic potential and leading to different effects such as the modification of electrical resistivity [16]. Moreover, the role of disorder is gaining attention in the study of advanced materials such as high-entropy alloys (HEA), consisting of multiple elements mixed in near-equal proportions that exhibit a high degree of configurational disorder. This disorder can lead to enhanced mechanical properties, such as increased strength and toughness, due to the complex interaction of different atomic species, which hinders dislocation motion and crack propagation [17,18]. In amorphous materials, the absence of periodicity leads to phenomena such as Anderson localization where the system can change from conductor to insulator as disorder increases [19]. The effects of disorder are also significant in the context of magnetic materials. In spin glasses, for example, the random distribution of ferromagnetic and antiferromagnetic interactions leads to frustration, where not all magnetic interactions can be satisfied simultaneously. This results in a highly degenerate ground state with a complex, non-trivial magnetic ordering, characterized by slow relaxation and memory effects [7,20]. Finally, the random arrangement of atoms or molecules in glasses can contribute to the thermal and mechanical properties of the material, such as its low thermal conductivity and high fracture toughness [21]. Mechanical forces, which can also exhibit randomness, further complicate phase transformations. As a matter of fact, the intensity of forces is critical in understanding processes at small scales where they may compete with the effects of thermal fluctuations. Random forces are a fundamental aspect of polymer unfolding, influencing the pathways, kinetics, and thermodynamics of the process. When polymers are subjected to mechanical stress, such as stretching or pulling, the presence of random forces arising from environmental heterogeneities or molecular interactions can significantly impact the folding/unfolding conformational transition. These effects are particularly evident in single-molecule force spectroscopy experiments, where individual proteins are subject to controlled mechanical forces. In this case the resulting force–extension curves often exhibit characteristics, such as force peaks and intermediate plateaus, which are directly influenced by thermal fluctuations, applied force fluctuations, and disorder in the macromolecule structure. This corresponds to a complex energy landscape that can be modified by the randomness affecting the barriers separating different configurations or inducing the formation of metastable states that can act as intermediate steps in the unfolding process [22,23,24,25].

In order to investigate the role, interplay, and competition of the environment temperature and the disorder in the external forces, in this paper, we consider a prototypical one-dimensional system where the units interact by a two-well elastic potential (nearest-neighbor interaction). We also include long-range energy terms modeled as Ising interactions. These non-local interactions among the elements favor the creation or suppression of domain walls with the nucleation and propagation of interfaces in the material. Our approach is based on the use of spin variables, a methodology that has been recently used to analyze folded/unfolded conformational changes in macromolecules, denaturation phenomena in nucleic acids, and decohesion phenomena from a substrate [26,27,28,29,30,31,32,33]. In these contexts, each value of the variable corresponds to a different phase. Here, we consider an extension of the model adding the effect of disorder in the force applied on a system of multi-stable elements. In particular, this disorder is represented as a random force acting on each element with a Gaussian probability distribution characterized by a fixed average and standard deviation. The model of the system is then mapped on the Random Field Ising Model (RFIM) [34,35,36,37,38,39,40,41,42,43,44,45,46]. Thus, we analyze the system in the thermodynamical limit by obtaining the expectation value (averaged on the quenched disorder) and the self-consistent equation for the order parameter by making use of the replica approach introduced in the context of the study of spin glasses [7]. By using the methods of Statistical Mechanics, in Section 2, we obtain the partition function for assigned random forces, and we derive the (disorder-averaged) force–displacement relation. In Section 3, we then analyze the phenomenology for a system with a small number of elements. We obtain numerical evidence of the effect of the thermal fluctuations, interaction strength among the spins, and the role of the force fluctuations among different realizations. In Section 4, we justify these numerical results in the thermodynamical limit by considering the case of quenched disorder [7] and evaluating the partition function, the free energy, and the self-consistent equation for the order parameter and phase diagram of the model. In particular, we obtain that even in the limit of zero temperature, the ordered phase can be destroyed by the disorder in the force. At the same time, in the limit of small force fluctuations, it is possible to identify, in the framework of replica symmetric solutions, the critical temperature between ordered and disordered configurations. In terms of the force–strain relations, this corresponds to the disappearance of the Maxwell line between the different elastic branches of the system. Finally, in Section 5, we draw the conclusions and foresee possible extensions.

## 2. Model

Consider a one-dimensional system of N+1 units interacting via nearest-neighbor bistable springs. Each spring link is characterized by a two-well energy, corresponding to two different material phases. In order to properly introduce the thermodynamics on non-convex, non-local systems, we adopt a method based on spin variables, by extending classical one-dimensional schemes to consider the fundamental effect of stiffness and elasticity of the different states. Thus, we introduce a series of discrete (spin) variables, which are able to identify the phase of the units. In so doing, with the aim of attaining analytical results, we can consider two separated and different quadratic functions each representing a well of the potential energy, instead of more complicated bistable energy densities. The introduction of the spin variables strongly simplifies the calculation of the partition functions and consequently, the analysis of the macroscopic thermodynamic quantities. Indeed, in order to compute the partition function, we sum over the spin variables, and we integrate the classical continuous variables (thus obtaining a classical spin model approach but with the important effect of the deformability of the domains due to the elastic energy). This simplification let us obtain analytical solutions as compared with the numerical results. This is important both for the interpretation of the thermo-mechanical effect and for deducing macroscale homogenized effects of a continuum material constituted by unfolding or breakable macromolecules. We can thus derive analytical formulas for the partition function, free energy, and the relation between the fixed applied force and total average strain. On the other hand, the presence of the disorder makes the analysis more complicated. As a matter of fact, the presence of a different force acting on each element, sampled from a normal distribution characterized by an average *f* and a standard deviation σ, does not allow us to derive a closed simple form of the constitutive relation between force and average strain. At the same time, it is possible to obtain an expression in terms of average values over the disorder probability density function.

**Figure 1:**
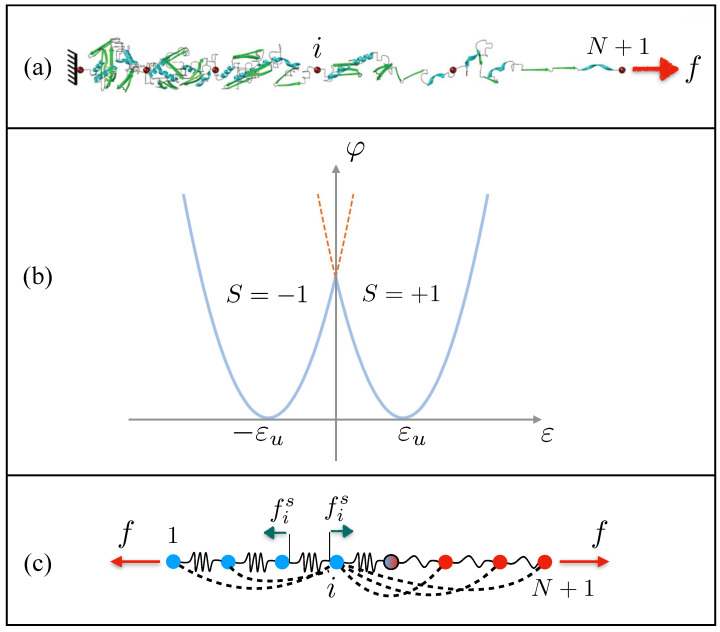
(**a**) System of phase changing domains subjected to an external force *f* acting on one end of the chain. (**b**) Bi-parabolic energy of a single spring. (**c**) Scheme of the interactions acting on the elements of the chain: nearest-neighbor units interact by elastic springs (local interaction, continuous lines), all units interact via non-local energy terms allowing long range interactions (dashed lines).

The aim of the model is the description of the joint effect of disorder and temperature on the transformation between different phases of each domain of the material system subjected to an assigned force acting on one end. Thus, we may think of a chain of units undergoing a conformational transition under the effect of an external force (see Figure 1a). Each spring is characterized by a bi-parabolic energy (see Figure 1b)

(1) $$\varphi =\frac{kl}{2}{\left(\varepsilon -\varepsilon_{u}\;S\right)}^{2},$$

where ε is the spring strain, *l* its natural length, *k* its stiffness, and S={−1,1} the internal discrete variable denoting the different phases (so that $-\varepsilon_{u}$ and $+\varepsilon_{u}$ represent the reference strains of the first and second well, respectively). We considered springs with the same value of stiffness *k* in both branches. Moreover, the minimum of both energy wells was set to zero. The generalization to more general cases is straightforward. In more detail, since the separated wells are represented by quadratic terms, the integration to obtain the partition function can be performed straightforwardly since it acts on Gaussian functions.

The total energy (including the force *f* acting on the final element) is

(2) $$\phi =\frac{kl}{2}\sum_{i=1}^{N}{\left(\varepsilon_{i}-\varepsilon_{u}\;S_{i}\right)}^{2}-\frac{J}{N}\sum_{i,j}S_{i}S_{j}-fl\sum_{i=1}^{N}\varepsilon_{i}.$$

In the following, without loss of generality, we consider l=1. In the energy, we include non-local interaction terms. In particular, these energetic terms are used to introduce the effect of interfaces between domains that exhibit different phases, thus allowing us to describe fundamental features of a material at the microscale. In particular, by following [31], we introduce the non-local, long-range, interaction by using an Ising-type model penalizing the interface formation, i.e., considering J>0, corresponding to favoring phase coalescence (ferromagnetic case). We notice that the Ising long-range interaction allows each unit to interact with all other elements of the system. This allows us to consider a mean-field approximation in the thermodynamical limit. These terms approximate the presence of non-local springs if the non-local terms have negligible stiffness as compared to local terms [47]. In that case, it is possible to show that the non-local energy term can be simplified by assuming that the lengths of the units are approximated by the equilibrium lengths of the explored wells and the behavior of the system is described by the classical Ising interaction. The solution of this problem can be found in [27] without non-local effects and in [28] for non-local effects. The case with non-identical wells is studied in [31]. We notice that the interaction term in Equation (2) allows the possibility that different domains can interact even if at large distance. This approach allows the use of the mean-field approximation in the thermodynamical limit N→∞.

Here, we generalize the previous model by assuming that, besides the elastic force acting on each spring and the external force *f*, there is an external random force $f_{i}^{s}$ due to the action of the environment. In particular, we assume that the force $f_{i}^{s}$ acting on the *i*th spring is assigned as a random variable with 0 average value and variance $\sigma^{2}$ of a Gaussian probability distribution

(3) $$p\left(f_{i}^{s}\right)=\frac{1}{\sqrt{2\pi \sigma^{2}}}e^{-\frac{{\left(f_{i}^{s}\right)}^{2}}{2\sigma^{2}}}.$$

It is important to point out that here, we consider the case of *quenched* disorder. In other words, we consider *slow* noise [7] characterized by time scales much larger than the ones regulating loading rate and relaxation to the equilibrium. As a consequence, we evaluate the statistical averages of physical observables at fixed disorder, and then we average the quantities over the force probability distribution. Thus, we consider the energy (depending on the realization of the disorder)

(4) $$\phi \left(\left\{f_{i}\right\},\left\{S_{i}\right\}\right)=\frac{k}{2}\sum_{i=1}^{N}{\left(\varepsilon_{i}-\varepsilon_{u}\;S_{i}\right)}^{2}-\frac{J}{N}\sum_{i,j}S_{i}S_{j}-\sum_{i=1}^{N}f_{i}\varepsilon_{i},$$

where

(5) $$f_{i}:=f+f_{i}^{s},$$

represents the total force acting at equilibrium on the *i*th spring.

We can evaluate the partition function $Z(\left\{f_{i}^{s}\right\})$ for a fixed configuration of forces $\left\{f_{i}^{s}\right\}$ as

(6) $$\begin{array}{ccc} Z & = & Z(\left\{f_{i}\right\})\\ & = & \sum_{\left\{S_{i}\right\}}\int_{R^{N}}\prod_{i}d\varepsilon_{i}e^{-\beta \phi }=\sum_{\left\{S_{i}\right\}}e^{\beta \frac{J}{N}\sum_{i,j}S_{i}S_{j}}\int_{R^{N}}\prod_{i}d\varepsilon_{i}e^{-\beta [\frac{k}{2}\sum_{i=1}^{N}{\left(\varepsilon_{i}-\varepsilon_{u}\;S_{i}\right)}^{2}-\sum_{i=1}^{N}f_{i}\varepsilon_{i}]}, \end{array}$$

where $\beta =k_{B}/T$, with $k_{B}$ the Boltzmann constant. In the following, we adopt the convention of setting $k_{B}=1$. By the classical Gaussian integration, we obtain

(7) $$Z={\left(\frac{2\pi \varepsilon_{u}^{2}}{\beta \;k}\right)}^{N/2}e^{\frac{\beta }{2k}\sum_{i=1}^{N}f_{i}^{2}}\sum_{\left\{S_{i}\right\}}e^{\beta (\frac{J}{N}\sum_{i,j}S_{i}S_{j}+\sum_{i=1}^{N}f_{i}S_{i})},$$

where, with a slight abuse of notation, we performed the substitution $\beta \varepsilon_{u}\to \beta$, $k\varepsilon_{u}\to k$, $J/\varepsilon_{u}\to J$. In order to evaluate the relation of the applied force and the strain of the system, we can compute the expectation value of $\varepsilon_{i}$ and its dependence on the fluctuating force $f_{i}^{s}$ (with variance $\sigma^{2}$) and temperature. Using the same abuse of notation, we find

(8) $$\begin{array}{ccc} \langle \varepsilon_{i}\rangle & = & \frac{1}{Z}\sum_{\left\{S_{i}\right\}}e^{\beta \frac{J}{N}\sum_{i,j}S_{i}S_{j}}\int_{R}d\varepsilon_{i}\varepsilon_{i}e^{-\beta [\frac{k}{2}{\left(\varepsilon_{i}-\varepsilon_{u}S_{i}\right)}^{2}-f_{i}\varepsilon_{i}]}\prod_{i\neq j}\int_{R}d\varepsilon_{j}e^{-\beta [\frac{k}{2}{\left(\varepsilon_{j}-\varepsilon_{u}S_{j}\right)}^{2}-f_{j}\varepsilon_{j}]}\\ & = & \frac{f}{k}+\frac{f_{i}^{s}}{k}+\varepsilon_{u}\langle S_{i}\rangle , \end{array}$$

where we introduced the phase state expectation value for the *i*th element

(9) $$\langle S_{i}\rangle =\frac{\sum_{\left\{S_{i}\right\}}S_{i}e^{\beta (\frac{J}{N}\sum_{i,j}S_{i}S_{j}+\sum_{i=1}^{N}f_{i}S_{i})}}{\sum_{\left\{S_{i}\right\}}e^{\beta (\frac{J}{N}\sum_{i,j}S_{i}S_{j}+\sum_{i=1}^{N}f_{i}S_{i})}}.$$

Using Equation (8), the expectation value of the overall strain $\bar{\varepsilon }=\frac{1}{N}\sum_{i}\langle \varepsilon_{i}\rangle$ reads

(10) $$\bar{\varepsilon }=\frac{\bar{f}}{k}+\varepsilon_{u}\bar{S},$$

where we considered the mean values of the random force and of the spin variable

(11) $$\bar{f}=f+\frac{1}{N}\sum_{i=1}^{N}f_{i}^{s},\;\bar{S}=\frac{1}{N}\sum_{i=1}^{N}\langle S_{i}\rangle .$$

Finally, the disorder-averaged force–strain relation takes the form

(12) $$\frac{f}{k}={\langle \bar{\varepsilon }\rangle }_{s}-\varepsilon_{u}{\langle \bar{S}\rangle }_{s},$$

where ${\langle .\rangle }_{s}$ denotes the disorder-averaged quantities obtained by using Equation (3).

In the following sections, we analyze this formula numerically for a small system and by an analytical computation based on the replica method in the thermodynamical limit [7].

## 3. Effects of Disorder and Temperature for a Small System: Numerical Results

In order to study the effects of disorder and temperature (as well as their interplay), we performed a numerical analysis for a small system based on Equations (10)–(12). In particular, we fixed the size of the system and sampled a large number of configurations for the independent random forces acting on the system (fixing *f* and σ). For each realization of the random forces, we computed $\bar{S}$ and thus $\bar{\varepsilon }$ by using Equations (9)–(11). Finally, we computed ${\langle \bar{\varepsilon }\rangle }_{s}$ over the sampled configurations for fixed values of *f* and σ.

**Figure 2:**
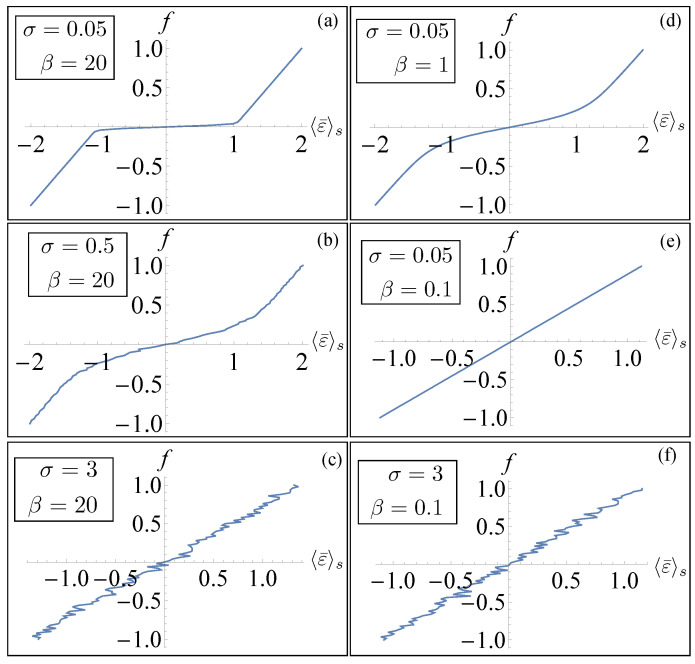
Numerical results of the relation between force and disorder-averaged strain for a system made of n=6 units. In the panels we chose the results for different values of the rescaled inverse temperature β and the standard deviation σ of the probability density of the random force $f_{i}^{s}$ applied to each element of the system. (**a**–**f**) We fixed the values J=1, k=1, and $\varepsilon_{u}=1$. The average of ${\langle \bar{\varepsilon }\rangle }_{s}$ was evaluated over $10^{3}$ configurations for each value of *f*. The procedure for the computation of ${\langle \bar{\varepsilon }\rangle }_{s}$ is explained in the main text.

**Figure 3:**
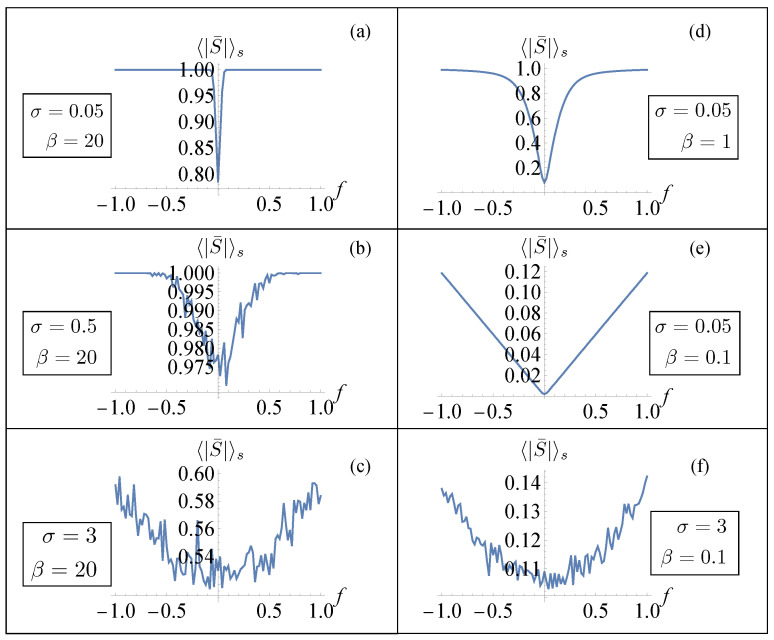
Numerical results (for a system made of n=6 units) of the relation between the average force *f* and of the disorder-averaged absolute value of the mean value of the spin variable $\langle |\bar{S}{|\rangle }_{s}$. (**a**–**f**) We fixed the values J=1, k=1, and $\varepsilon_{u}=1$.

**Figure 4:**
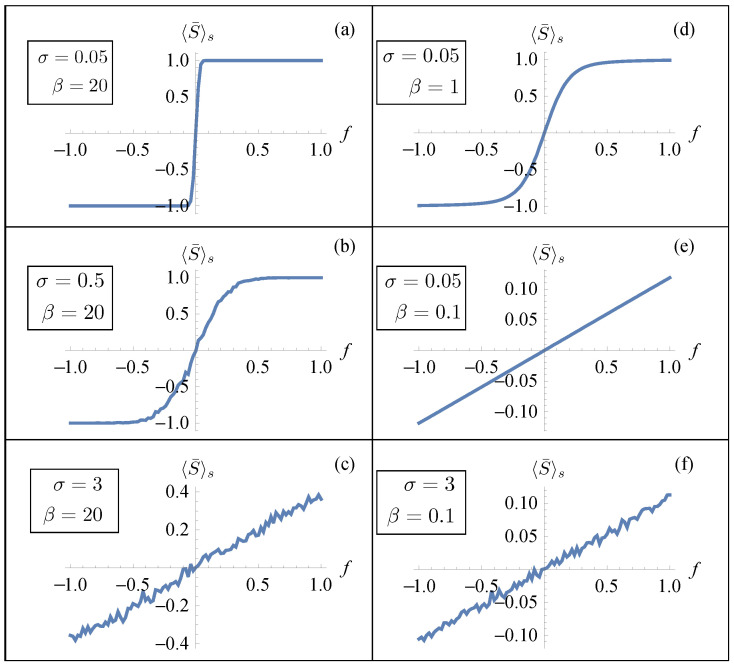
Numerical results (for a system made of n=6 units) of the relation between the average force *f* and of the disorder-averaged mean value of the spin variable ${\langle \bar{S}\rangle }_{s}$. (**a**–**f**) We fixed the values J=1, k=1, $\varepsilon_{u}=1$.

In Figure 2, we plotted the results for a system of n=6 elements. In the left panels, we exhibit the important effect of noise in inducing a non-cooperative transition behavior. In these figures, we considered a small value of *T* (large β) so that thermal fluctuations were negligible and the effect of noise was enhanced by increasing σ. Thus, large values of disorder were able to suppress the sharp transition between the different phases. A similar effect was obtained, as shown in panels (d) and (e), by considering a small value of σ and increasing the temperature *T*. In panel (f), we considered the overall effect of considering large values of temperature and σ. This behavior was consistent with a previous analysis performed in the absence of disorder, where the role of the temperature was highlighted [28]. In particular, in the zero-temperature limit (mechanical limit), we observed two elastic branches connected by a horizontal line (Maxwell line) corresponding to a fully cooperative phase transformation of the system. On the other hand, the effect of temperature modified the behavior of the force–strain diagram with an increase in the slope of the line connecting the elastic branches. From Equation (12), we can deduce that this behavior is associated with the dependence of ${\langle \bar{S}\rangle }_{s}$ on σ and β. In order to verify this observation, in Figure 3, we show the numerical evaluation of the dependence of the disorder-averaged absolute value of the mean of the spin variable $\langle |\bar{S}{|\rangle }_{s}$ on the mean force *f*. We evaluated the absolute value in order to avoid cancellations in the average and observed if the expectation value of that quantity was drastically different from the value one. We observed that in the almost ideal (small σ) low-temperature (large β) case (panel (a)) the value of $\langle |\bar{S}{|\rangle }_{s}$ was always very close to one (or equal to one) for all values of *f* indicating a cooperative phase transition. On the other hand, larger values of σ corresponded to a decrease in $\langle |\bar{S}{|\rangle }_{s}$ at a fixed *f* indicating a non-cooperative transition between the phases with the sharp transition between the two phases no longer observable. For a comparison, in Figure 4, we show the numerical evaluation of the dependence of the disorder-averaged mean value of the spin variable ${\langle \bar{S}\rangle }_{s}$ on *f*. In this case, for small values of *f*, the cancellations in the average did not allow us to observe a value of this quantity different from zero for small values of temperature and strength of the disorder.

From these numerical results, we can deduce that there is a non-trivial interplay between temperature and disorder, collectively contributing to the destruction of a clear bistable behavior of the system. In order to get more insight into these numerical results and obtain quantitative information about the force–strain relation for a large system, in the following section we make use of the approach based on the replica method. That way, we can obtain a phase diagram for the system and justify the previously obtained results.

## 4. Effects of Disorder and Temperature in the Thermodynamical Limit: Replica Method Approach

In order to obtain expectation values of physical observables, one has to compute the value of the free energy averaged over the disorder due to the random forces $f_{i}^{s}$. Thus, we need to evaluate

(13) $${\langle G\rangle }_{s}=-\frac{1}{\beta }{\langle \ln Z\rangle }_{s},$$

where, as before, ${\langle .\rangle }_{s}$ denotes the disorder average obtained by using Equation (3). As a consequence, the presence of randomness in the force term suggests we should approach the problem of evaluating the free energy by using the techniques developed for complex systems based on the replica method. The so-called replica trick is a mathematical method used in Statistical Mechanics to obtain information about the free energy and other physical observables for complex and disordered systems [7]. In this context, the approach (and its extensions) helps in treating spin glasses, where the direct analytical computation of the disorder-averaged free energy is intractable. By introducing replicas and averaging over the disorder (before taking the replica limit), one can obtain results about the free-energy landscape and the presence and features of phase transitions [7]. An important comment is in order. Strikingly, the structure of the partition function in Equation (7) is formally similar to the case of the so-called Random Field Ising Model (RFIM), where the coupling among the spins is constant, and the external field linearly coupled with each spin is a random variable [34,35,36,37,38,39,40,41,42,43,44,45,46]. In our case we also observe that the RFIM is generalized by the presence of the elastic terms. Thus, in the following, we analyze how the replica-based approach can be used to deduce the randomness-dependent expectation values even in presence of elastic energy contributions.

### 4.1. Application of the Replica Method to the Model

We start from the identity

(14) $$\ln Z=\lim_{n\to 0}\frac{Z^{n}-1}{n}=\lim_{n\to 0}\frac{\partial Z^{n}}{\partial n},$$

where *n* denotes the replica number. Due to this result, the evaluation of the disorder average ${\langle \ln Z\rangle }_{s}$ can be reduced to the computation of ${\langle Z^{n}\rangle }_{s}$. Thus, from Equation (7), we obtain

(15) $$Z^{n}={\left(\frac{2\pi \varepsilon_{u}^{2}}{\beta \;k}\right)}^{Nn/2}e^{\frac{n\beta }{2k}\sum_{i=1}^{N}f_{i}^{2}}\sum_{\left\{S_{i}^{\alpha }\right\}}e^{\beta (\frac{J}{N}\sum_{\alpha =1}^{n}\sum_{i,j=1}^{N}S_{i}^{\alpha }S_{j}^{\alpha }+\beta \sum_{\alpha =1}^{n}\sum_{i=1}^{N}f_{i}S_{i}^{\alpha })},$$

where α is the replica index. Using the properties of Gaussian integrals, we can now evaluate ${\langle Z^{n}\rangle }_{s}$ as

(16) $$\begin{array}{ccc} {\langle Z^{n}\rangle }_{s} & = & \int_{R^{N}}\prod_{i}df_{i}^{s}\prod_{i}p\left(f_{i}^{s}\right)Z^{n}\\ & = & {\left(\frac{2\pi \varepsilon_{u}^{2}}{\beta \;k}\right)}^{Nn/2}{\left(\frac{k}{k-n\beta \sigma^{2}}\right)}^{N/2}e^{\frac{\beta }{2}\frac{nNf^{2}}{k-n\beta \sigma^{2}}}\\ & \times & \sum_{\left\{S_{i}^{\alpha }\right\}}e^{\beta \frac{J}{N}\sum_{\alpha =1}^{n}\sum_{i,j=1}^{N}S_{i}^{\alpha }S_{j}^{\alpha }+\beta f\frac{k}{k-n\beta \sigma^{2}}\sum_{i=1}^{N}\sum_{\alpha =1}^{n}S_{i}^{\alpha }+\frac{\beta^{2}\sigma^{2}}{2}\frac{k}{k-n\beta \sigma^{2}}\sum_{i=1}^{N}{\left(\sum_{\alpha =1}^{n}S_{i}^{\alpha }\right)}^{2}}, \end{array}$$

where we have imposed the condition $k-n\beta \sigma^{2}>0$ in order to ensure the convergence of the Gaussian integrals. This condition is automatically fulfilled in the limit n→0. We can now use the identity

(17) $$\sum_{\alpha =1}^{n}\sum_{i,j=1}^{N}S_{i}^{\alpha }S_{j}^{\alpha }=\sum_{\alpha =1}^{n}{\left(\sum_{i=1}^{N}S_{i}^{\alpha }\right)}^{2},$$

and the Hubbard–Stratonovich transformation

(18) $$e^{\beta \frac{J}{N}\sum_{\alpha =1}^{n}{\left(\sum_{i=1}^{N}S_{i}^{\alpha }\right)}^{2}}=\frac{1}{{\left(2\pi \right)}^{n/2}}\int_{R^{n}}\prod_{\alpha =1}^{n}dy_{\alpha }e^{\sum_{\alpha =1}^{n}\left[y_{\alpha }\sqrt{\frac{2\beta J}{N}}\sum_{i=1}^{N}S_{i}^{\alpha }-\frac{1}{2}y_{\alpha }^{2}\right]},$$

so that

(19) $$\begin{array}{c} {\langle Z^{n}\rangle }_{s}=\frac{C_{n}}{{\left(2\pi \right)}^{n/2}}\sum_{\left\{S_{i}^{\alpha }\right\}}\int_{R^{n}}\prod_{\alpha =1}^{n}dy_{\alpha }e^{-\frac{1}{2}\sum_{\alpha =1}^{n}y_{\alpha }^{2}}\;e^{g(\left\{S_{i}^{\alpha }\right\},\left\{y_{\alpha }\right\})}, \end{array}$$

where

(20) $$\begin{array}{ccc} g(\left\{S_{i}^{\alpha }\right\},\left\{y_{\alpha }\right\}) & = & \sqrt{\frac{2\beta J}{N}}\sum_{i=1}^{N}\sum_{\alpha =1}^{n}y_{\alpha }S_{i}^{\alpha }+\beta f\frac{k}{k-n\beta \sigma^{2}}\sum_{i=1}^{N}\sum_{\alpha =1}^{n}S_{i}^{\alpha }\\ & + & \frac{\beta^{2}\sigma^{2}}{2}\frac{k}{k-n\beta \sigma^{2}}\sum_{i=1}^{N}{\left(\sum_{\alpha =1}^{n}S_{i}^{\alpha }\right)}^{2}, \end{array}$$

and

(21) $$C_{n}={\left(\frac{2\pi \varepsilon_{u}^{2}}{\beta \;k}\right)}^{Nn/2}{\left(\frac{k}{k-n\beta \sigma^{2}}\right)}^{N/2}e^{\frac{\beta }{2}\frac{nNf^{2}}{k-n\beta \sigma^{2}}}.$$

By using the rescaling $\eta_{\alpha }=y_{\alpha }/\sqrt{N}$, we can rewrite ${\langle Z^{n}\rangle }_{s}$ as

(22) $$\begin{array}{c} {\langle Z^{n}\rangle }_{s}=C_{n}{\left(\frac{N}{2\pi }\right)}^{n/2}\int \prod_{\alpha =1}^{n}d\eta_{\alpha }e^{-\frac{N}{2}\sum_{\alpha =1}^{n}\eta_{\alpha }^{2}}{\left[Z_{1}\left(\eta_{\alpha }\right)\right]}^{N}, \end{array}$$

where

(23) $$\begin{array}{c} Z_{1}\left(\eta_{\alpha }\right)=\sum_{\left\{S^{\alpha }\right\}}e^{\left[\sqrt{2\beta J}\sum_{\alpha =1}^{n}\eta_{\alpha }S^{\alpha }+\beta f\frac{k}{k-n\beta \sigma^{2}}\sum_{\alpha =1}^{n}S^{\alpha }+\frac{\beta^{2}\sigma^{2}}{2}\frac{k}{k-n\beta \sigma^{2}}{\left(\sum_{\alpha =1}^{n}S^{\alpha }\right)}^{2}\right]} \end{array}$$

is the partition function of the replicas acting on one element of the chain.

### 4.2. Replica Symmetric Solution in the Thermodynamical Limit

In the thermodynamic limit N→+∞, we can use the saddle-point method. In particular, we consider the replica symmetric solution where $\eta_{\alpha }=\eta$ for all α. From Equation (22), we can see that the saddle point is obtained by the equation

(24) $$\begin{array}{c} \frac{\partial }{\partial \eta }\left[-\frac{n\eta^{2}}{2}+\ln Z_{1}\left(\eta \right)\right]=0, \end{array}$$

corresponding to a solution $\bar{\eta }$ that verifies the self-consistent equation

(25) $$\begin{array}{c} n\bar{\eta }=\frac{\sqrt{2\beta J}}{Z_{1}\left(\bar{\eta }\right)}\sum_{\left\{S^{\alpha }\right\}}\left(\sum_{\alpha =1}^{n}S^{\alpha }\right)e^{\Omega (S^{\alpha },\bar{\eta })}, \end{array}$$

with

(26) $$\begin{array}{c} \Omega \left(S^{\alpha },\bar{\eta }\right)=\sqrt{2\beta J}\bar{\eta }\sum_{\alpha =1}^{n}S^{\alpha }+\beta f\frac{k}{k-n\beta \sigma^{2}}\sum_{\alpha =1}^{n}S^{\alpha }+\frac{\beta^{2}\sigma^{2}}{2}\frac{k}{k-n\beta \sigma^{2}}{\left(\sum_{\alpha =1}^{n}S^{\alpha }\right)}^{2}. \end{array}$$

The value of $\bar{\eta }$ is obtained by solving the following equation

(27) $$\begin{array}{c} m=\frac{\bar{\eta }}{\sqrt{2\beta J}}=\frac{1}{Z_{1}\left(m\right)}\sum_{\left\{S^{\alpha }\right\}}\left(\frac{1}{n}\sum_{\alpha =1}^{n}S^{\alpha }\right)e^{\Omega (S^{\alpha },m)}, \end{array}$$

where

(28) $$\begin{array}{ccc} Z_{1}\left(m\right) & = & \sum_{\left\{S^{\alpha }\right\}}e^{\Omega (S^{\alpha },\bar{\eta })}, \end{array}$$

(29) $$\begin{array}{ccc} \Omega (S^{\alpha },m) & = & 2\beta Jm\sum_{\alpha =1}^{n}S^{\alpha }+\beta f\frac{k}{k-n\beta \sigma^{2}}\sum_{\alpha =1}^{n}S^{\alpha }+\frac{\beta^{2}\sigma^{2}}{2}\frac{k}{k-n\beta \sigma^{2}}{\left(\sum_{\alpha =1}^{n}S^{\alpha }\right)}^{2}. \end{array}$$

Thus, *m* represents the average phase parameter obtained from the replicas on a site of the chain. We can evaluate ${\langle Z^{n}\rangle }_{s}$ in the thermodynamic limit with the replica symmetric solution in the saddle-point approximation as

(30) $$\begin{array}{c} {\langle Z^{n}\rangle }_{s}≃C_{n}{\left(\frac{N}{2\pi }\right)}^{n/2}e^{-Nn\beta Jm^{2}}Z_{1}{\left(m\right)}^{N}. \end{array}$$

Finally, we can write

(31) $$\begin{array}{ccc} Z_{1}\left(m\right) & = & \frac{1}{\sqrt{2\pi }}\int_{R}dxe^{-\frac{x^{2}}{2}}\prod_{\alpha =1}^{n}\sum_{S^{\alpha }=-1,1}e^{\left(x\sqrt{\beta^{2}\sigma^{2}\frac{k}{k-n\beta \sigma^{2}}}+2\beta Jm+\beta f\frac{k}{k-n\beta \sigma^{2}}\right)S^{\alpha }}\\ & = & \frac{1}{\sqrt{2\pi }}\int_{R}dxe^{-\frac{x^{2}}{2}}{\left[2\cosh\left(x\sqrt{\beta^{2}\sigma^{2}\frac{k}{k-n\beta \sigma^{2}}}+2\beta Jm+\beta f\frac{k}{k-n\beta \sigma^{2}}\right)\right]}^{n}. \end{array}$$

An analogous calculation for the self-consistent equation gives

(32) $$\begin{array}{ccc} m & = & \frac{1}{\sqrt{2\pi }Z_{1}\left(m\right)}\int_{R}dxe^{-\frac{x^{2}}{2}}{\left[2\cosh\left(x\sqrt{\beta^{2}\sigma^{2}\frac{k}{k-n\beta \sigma^{2}}}+2\beta Jm+\beta f\frac{k}{k-n\beta \sigma^{2}}\right)\right]}^{n}\\ & \times & \tanh\left(x\sqrt{\beta^{2}\sigma^{2}\frac{k}{k-n\beta \sigma^{2}}}+2\beta Jm+\beta f\frac{k}{k-n\beta \sigma^{2}}\right). \end{array}$$

Form Equation (14), we can evaluate ${\langle G\rangle }_{s}$ as

(33) $${\langle G\rangle }_{s}=-\frac{1}{\beta }\lim_{n\to 0}\frac{\partial {\langle Z^{n}\rangle }_{s}}{\partial n}.$$

By considering $x=\frac{f^{s}}{\sigma }$, we obtain

(34) $$\begin{array}{ccc} {\langle G\rangle }_{s} & = & -N\frac{k}{2}\left(f^{2}+\sigma^{2}\right)\\ & + & N\left\{Jm^{2}-\frac{1}{\beta }\int_{R}df^{s}p\left(f^{s}\right)\ln\left[2\cosh\left(2\beta Jm+\beta (f+f^{s})\right)\right]\right\}-\frac{1}{2\beta }\ln\lambda , \end{array}$$

where

(35) $$\lambda =\frac{N}{2\pi }{\left(\frac{2\pi \varepsilon_{u}^{2}}{k\beta }\right)}^{N/2}$$

and *m* is given (considering the limit n→0) by

(36) $$m=\int_{R}df^{s}p\left(f^{s}\right)\tanh\left(2\beta Jm+\beta (f+f^{s})\right).$$

We can find the condition for the transition between a disordered (m=0) and ordered phase (m≠0) from Equation (36) by imposing that

(37) $$\frac{d}{dm}{\left[\int_{R}df^{s}p\left(f^{s}\right)\tanh\left(2\beta Jm+\beta (f+f^{s})\right)\right]}_{m=0}=1.$$

We then use the rescaling $\tilde{J}=J/\sigma$, $\tilde{\beta }=\beta \sigma$, $\tilde{f}=f\beta$, ${\tilde{f}}^{s}=f^{s}\beta$ and find the transition relation

(38) $$\frac{1}{2\tilde{J}}=\frac{1}{2}\frac{\sigma }{J}=\left[\int_{R}\frac{d{\tilde{f}}^{s}}{\sqrt{2\pi }}\frac{e^{-\frac{{\left({\tilde{f}}^{s}\right)}^{2}}{2{\tilde{\beta }}^{2}}}}{\cosh^{2}\left(\tilde{f}+{\tilde{f}}^{s}\right)}\right].$$

We also notice that if $\tilde{\beta }\to +\infty$ (which means zero temperature or infinite variance of the random force), the condition simplifies to

(39) $$\frac{1}{\tilde{J}}=\frac{\sigma }{J}=\sqrt{\frac{8}{\pi }}≃1.596,$$

and it is independent from the (average) rescaled applied force $\tilde{f}$. As previously pointed out, the obtained results have formal similarities with the RFIM.

**Figure 5:**
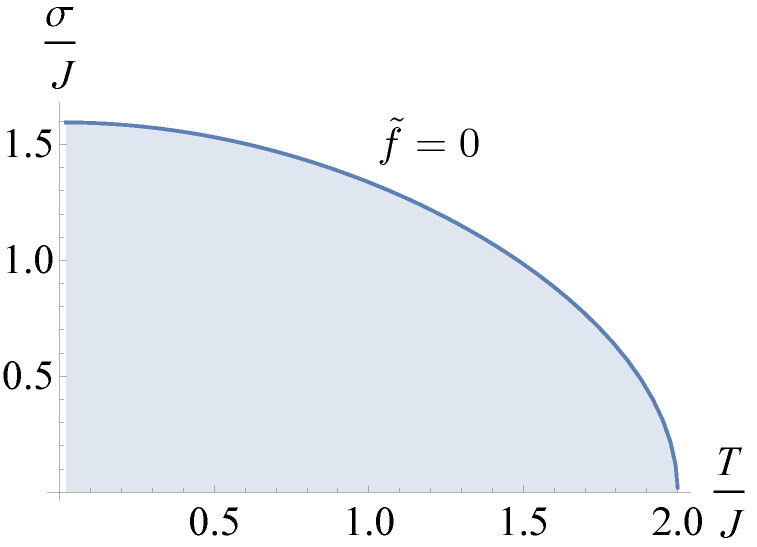
Phase diagram (mean value of the rescaled force $\tilde{f}=0$) between disordered and ordered phase (above and below the line, respectively).

In Figure 5, we plotted the phase diagram of the system (mean value of the rescaled force $\tilde{f}=0$) with ordered and disordered phase (above and below the line, respectively). In particular, for larger values of temperature and randomness, the system underwent a transition from the ordered to the disordered phase. As obtained in the discrete case, we observed the possibility of attaining the phase transition at small temperature by increasing the disorder or at small disorder by increasing the temperature. Interestingly, we were able to determine the combined effect thanks to the analytical results obtained above. The phase diagram allowed us to justify the numerical results obtained in Section 3. As a matter of fact, there, we observed a smearing of the transition between phases for values of σ and β that could be compared to Figure 5. In particular, we noticed that the suppression of the sharp transition was obtained for values of σ>1.596 (previously obtained) and T/J>2.

**Figure 6:**
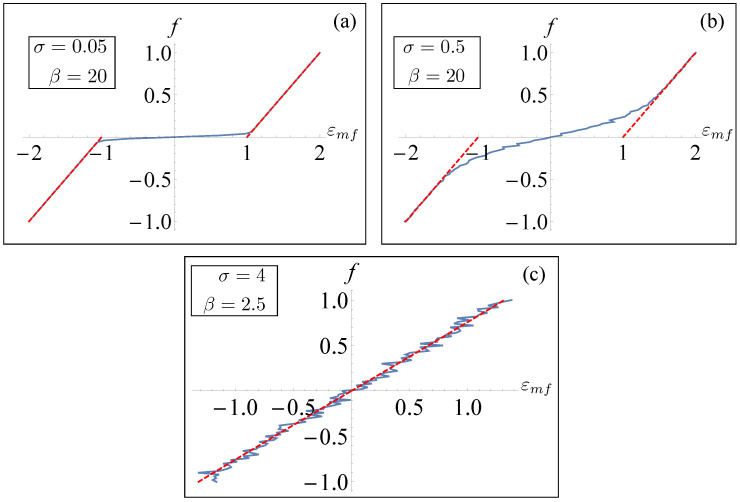
Relation between the mean-field strain and average applied force (dashed red) for different values of β and σ. For a comparison, we have included numerical results obtained for a small-size (N=6) system as described in Section 3.

In order to directly compare the results obtained in this section with the numerical results for a small system, we plotted the force–strain relation obtained in the mean-field approximation. In particular, we obtained *m* by solving numerically Equation (36) and defined the mean-field strain solution

(40) $$\varepsilon_{mf}=\frac{f}{k}+\varepsilon_{u}m,$$

that should be compared with the result obtained from Equation (12). In Figure 6, we considered this relation for different values of σ and $\beta =1/k_{B}T$, fixing $k_{B}=1$, k=1, $\varepsilon_{u}=1$, and J=1. We noticed that the behavior of the curves was clearly consistent with the T−σ phase diagram in Figure 5. For small values of temperatures (large values of β) and small values of σ the behavior displayed corresponded to a sharp transition between the two phases of the system, Figure 6a,b. On the other hand, even if the value of temperature was below the critical threshold, a large value of σ corresponded to a smearing of the bistable behavior of the system (Figure 6c). In order to compare the results, we superposed the curves obtained in Section 3 for a small-size system (numerical results) with the analytical curves. We observed that the behavior was consistent and thus, even if one must take into account the differences due to the small-size corrections, the analytical results in the thermodynamical limit can be used, as previously pointed out, to justify the numerical results obtained.

## 5. Conclusions

In this paper, we considered the problem of describing the phenomenon of phase transformation in a material in the presence of randomness in the external mechanical action. In particular, we investigated the effects of disorder and thermal fluctuations (and their interplay) in a prototypical one-dimensional model used to describe multi-stable materials. Through a combination of numerical results and analytical methods, we showed that the presence of disorder, here introduced in the form of fluctuations in the mechanical forces acting on each element of the system, fundamentally altered the response of the material.

In order to take into account the multi-stable behavior of the elastic springs, we used a discrete (spin) variable approach that was recently used in the context of phase transformations phenomena, conformational transition in macromolecular unfolding, and decohesion phenomena [26,27,28,29,30,31,32,33]. In order to go beyond the nearest-neighbor interaction of the units of the system, we considered the case of long-range interactions, responsible for the generation of walls between different phases, and used a mean-field approach to derive the solution in the thermodynamical limit. By using this methodology, the model could be naturally mapped onto a generalization of the Random Field Ising Model (RFIM) that also included elastic interactions.

In order to include the effect of disorder, we based the analysis on the so-called *replica method*. In particular, we showed that the approach could also be used in this case in order to obtain the analytical expression of the disorder-averaged free energy and force–strain relation in terms of the applied mean force and the expectation value of the mean strain. The replica trick allowed us to show that by considering long range interactions, it was possible to separate the role of the elastic energy and the generation of interfaces due to the spin interaction.

By numerical computation, we deduced the qualitative properties of the force–strain relation and its dependence on the temperature and standard deviation of the random forces’ probability distribution. We compared these results obtained for a small-scale system with the formulas obtained for a large system in the thermodynamic limit. That way, we were able to justify the numerical results in terms of the phase diagram for a large system, adapting the classical results related to the existence of a critical temperature and strength of the disorder (in terms of the standard deviation of the random force probability distribution) of the RFIM.

As a matter of fact, future work should aim to deepen our understanding of how different types of disorder interact with each other and with external fields (such as mechanical forces, thermal loads, and chemical reactions) to predict, control, and exploit phase transformations in technological applications. Additionally, the development of more sophisticated theoretical models that incorporate and allow one to study the interplay of disorder, external fields, and elastic interaction could provide further insight into the behavior of materials. The exploration of non-equilibrium dynamics and rate effects in disordered systems could also represent an important extension, particularly in materials where phase transformations occur far from equilibrium, such as in glasses [48].

In conclusion, the effects of disorder and randomness in a system that exhibits phenomena of phase transformations are profound and multifaceted. Our results, even applied in the context of long-range interaction allowing us to obtain analytical results including the effects of elastic interactions, indicate that disorder significantly impacts on the response of the system to external loading, for instance suppressing the critical behavior and playing a central role in determining the modified response of a material in view of real-world applications. It is possible to imagine generalizing the obtained results. In particular, one could consider a system with short-range interactions also including the disorder in the coupling constant of the interaction terms. From a technical point of view, in these cases, one should expect to be able to apply the methodology developed for spin glasses such as the replica symmetry-breaking technique and the cavity method [7]. These generalizations would be interesting for a comparison with experimental results and to study the role of engineered disorder to obtain novel features of a material and innovative responses to external action. The possibility of deducing fully analytical relations could result in a proper design of new materials and metamaterials.

## Data Availability

The data presented are available on request from the corresponding author.
